# Toosendanin Induces Hepatocyte Damage by Inhibiting Autophagic Flux via TFEB-Mediated Lysosomal Dysfunction

**DOI:** 10.3390/ph15121509

**Published:** 2022-12-03

**Authors:** Li Luo, Yonghong Liang, Yuanyuan Fu, Zhiyuan Liang, Jinfen Zheng, Jie Lan, Feihai Shen, Zhiying Huang

**Affiliations:** 1 School of Pharmaceutical Sciences, Sun Yat-sen University, Guangzhou 510006, China; 2 School of Pharmacy, Guangdong Pharmaceutical University, Guangzhou 510006, China

**Keywords:** toosendanin (TSN), hepatotoxicity, autophagy, autophagic flux, lysosome, TFEB

## Abstract

Toosendanin (TSN) is a triterpenoid from the fruit or bark of *Melia toosendan* Sieb et Zucc, which has clear antitumor and insecticidal activities, but it possesses limiting hepatotoxicity in clinical application. Autophagy is a degradation and recycling mechanism to maintain cellular homeostasis, and it also plays an essential role in TSN-induced hepatotoxicity. Nevertheless, the specific mechanism of TSN on autophagy-related hepatotoxicity is still unknown. The hepatotoxicity of TSN in vivo and in vitro was explored in this study. It was found that TSN induced the upregulation of the autophagy-marker microtubule-associated proteins 1A/1B light chain 3B (LC3B) and P62, the accumulation of autolysosomes, and the inhibition of autophagic flux. The middle and late stages of autophagy were mainly studied. The data showed that TSN did not affect the fusion of autophagosomes and lysosomes but significantly inhibited the acidity, the degradation capacity of lysosomes, and the expression of hydrolase cathepsin B (CTSB). The activation of autophagy could alleviate TSN-induced hepatocyte damage. TSN inhibited the expression of transcription factor EB (TFEB), which is a key transcription factor for many genes of autophagy and lysosomes, such as CTSB, and overexpression of TFEB alleviated the autophagic flux blockade caused by TSN. In summary, TSN caused hepatotoxicity by inhibiting TFEB-lysosome-mediated autophagic flux and activating autophagy by rapamycin (Rapa), which could effectively alleviate TSN-induced hepatotoxicity, indicating that targeting autophagy is a new strategy to intervene in the hepatotoxicity of TSN.

## 1. Introduction

Drug-induced liver injury (DILI) is a global, challenging liver disease and a major complication for many drugs, including chemical drugs, biologics, herbal medicines, and dietary supplements [1,2]. Due to differences in study design and the populations, inconsistent standards of definition, etc., DILI reportedly shows different epidemiology in different countries. The annual incidence of DILI is estimated to be 13.9/100,000 inhabitants in France [3], 19.1/100,000 inhabitants in Iceland [4], 2.4/100,000 inhabitants in Britain [5], 2.3/100,000 inhabitants in Sweden [6], 3.42/100,000 inhabitants in Spain [7], 12/100,000 inhabitants in South Korea [8], and 23.8/100,000 inhabitants in China [9]. However, the true incidence of DILI is difficult to estimate and may be much higher than reported. It is worth noting that the incidence of DILI caused by herbal medicine and dietary supplements (HDS) is increasing worldwide [1]. A national prospective study in South Korea showed that HDS were the main causes of liver injury, accounting for 27.5% of the inducing factors of DILI [8]. Nevertheless, the pathological mechanism of liver injury caused by most HDS is still unclear.

Toosendanin (TSN) is a tetracyclic triterpenoid extracted from the fruit or bark of *Melia toosendan* Sieb et Zucc [10]. In China, TSN is widely used as a commercialized and pesticide-free plant insecticide [11]. Extensive research has shown that TSN has a broad-spectrum anti-cancer effect by inhibiting proliferation and migration and reversing chemotherapeutic drug resistance in tumor cells [12,13]. TSN possesses anti-virus [14], anti-botulinum [15], and analgesic effects as well. However, recent evidence suggests that TSN or herbal medicines containing TSN caused hepatotoxicity, which limits its clinical application. TSN-induced hepatotoxicity was characterized by the elevation of serum alanine aminotransferase (ALT), aspartate aminotransferase (AST), and hepatocytes necrosis [16,17]). Previous research has established that TSN induced hepatocyte energy metabolism disorder, glutathione (GSH) depletion, mitochondrial dysfunction, and caspase activation [18]. However, the underlying mechanisms of TSN-induced hepatotoxicity remain unknown.

Autophagy is a cellular degradation and recycling mechanism that occurs in response to various stimuli, such as starvation and toxicity [19]. The whole dynamic process of autophagy is called autophagic flux, which can be blocked by suppressing autophagosome–lysosome fusion and inhibiting lysosomes’ degradation. Recent studies suggest that aberrant autophagic flux is involved in DILI, and autophagy possesses essential protective functions [20,21]. In most cases, autophagy plays a protective role in DILI by selectively removing damaged mitochondria, lipid droplets, protein aggregates, and adducts in hepatocytes [22]. For example, acetaminophen, a well-known drug with hepatotoxicity, caused autophagic flux blockade in mice livers. Pterostilbene had a protective effect against the liver injury caused by acetaminophen, by restoring autophagic flux, while autophagy inhibitor chloroquine (CQ) aggravated acetaminophen-induced liver injury [23]. Autophagy also plays a positive role in promoting hepatocyte survival in efavirenz- and alcohol-induced liver injury [24,25]. It has been reported that TSN caused liver injury in zebrafish, and the upregulation of *beclin1*, *autophagy-related gene (atg) 3*, and *microtubule-associated proteins 1A/1B light chain 3 (lc3)* gene indicated that autophagy participated in TSN-induced hepatotoxicity [26]. On the basis of these characteristics, we hypothesized that autophagy is involved in TSN-induced liver injury.

During autophagy, lysosomes participate in fusion with autophagosomes and the subsequent degradation of autophagic substrate. Disruption of lysosomal structure, the acidic environment, or lysosomal function would disturb autophagic flux. Abnormalities in lysosomal pH and cathepsin B (CTSB) protein expression level were observed in cadmium-induced liver injury [27]. Lysosomal dysfunction, specifically the downregulated expression of lysosomal membrane proteins 1 (LAMP1) and cathepsin D (CTSD), also played an important role in the hepatocyte autophagic flux blockade caused by 1,3-dichloro-2-propanol [28]. The effect of TSN on hepatocyte lysosomes remains elusive and needs further exploration.

Transcription factor EB (TFEB), one member of the microphthalmia/transcription factor E (MiT/TFE) family, is a key transcription factor for autophagy and lysosomal biogenesis. Under normal conditions, TFEB is phosphorylated by the mechanistic target of rapamycin kinase complex 1 (MTORC1) and remains inactive in the cytoplasm. Upon cellular stress, TFEB is dephosphorylated and translocated to the nucleus, specifically binding to the coordinated lysosomal enhancement and regulation (CLEAR) motif to regulate the transcriptional activation of autophagy and lysosome biogenesis genes [29]. Activation of TFEB-mediated autophagy by trehalose has been shown to attenuate mitochondrial dysfunction in cisplatin-induced acute kidney injury [30]. To understand the role of TFEB in TSN-induced hepatotoxicity, further research is warranted.

This study aims to further explore the role of autophagy in TSN-induced hepatotoxicity, with a focus on autophagic flux, particularly autophagosome–lysosome fusion and lysosomal degradation, as well as to elucidate the role of TFEB-mediated autophagy in TSN-induced hepatotoxicity.

## 2. Results

### 2.1. TSN Induces Cell Damage in HepG2 Cells and L02 Cells

**Figure 1:**
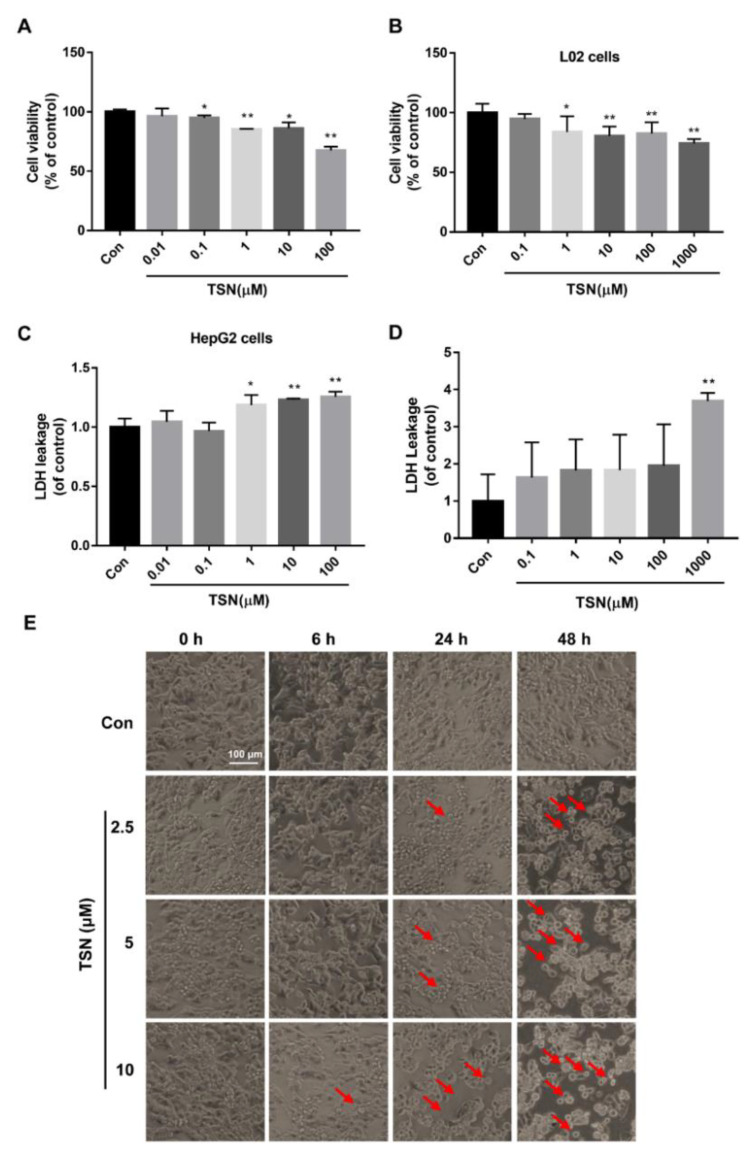
TSN induced cell damage in HepG2 and L02 cells. HepG2 and L02 cells were treated with TSN at various concentrations for 24 h. Cell viability of HepG2 cells (**A**) and L02 cells (**B**) was determined using CCK-8 assay kit. The cell culture supernatants of HepG2 cells (**C**) and L02 cells (**D**) were collected to measure LDH leakage using an LDH assay kit. (**E**) HepG2 cells were treated with TSN at various concentrations (2.5, 5, 10 µM) for different time durations (0, 6, 24, 48 h), and cellular morphology was captured under phase-contrast microscopy. Red arrows indicate the cells for which morphology changed, mainly shrinking and rounding. Scale bar: 100 µm. Data are presented as the mean ± SD of three independent experiments. * *p* < 0.05, ** *p* < 0.01 versus control.

To investigate the effect of TSN on the proliferation of HepG2 and L02 cells, cell viability was detected by a Cell Counting Kit-8 (CCK-8) assay. HepG2 cells were treated with TSN at various concentrations (0.01, 0.1, 1, 10, 100 µM) for 24 h. The viability of HepG2 cells with TSN exposure was reduced in a concentration-dependent manner (Figure 1A). Similar to HepG2 cells, the viability of L02 cells was also reduced with the increase in concentration of TSN exposure (Figure 1B). Lactate dehydrogenase (LDH), an intracellular and stable enzyme, rapidly releases into the extracellular environment when the cell is damaged, which is usually used to evaluate the integrity of plasma membrane [31]. To further verify the toxicity of TSN on hepatocytes, the cell culture supernatants of HepG2 and L02 cells treated with TSN were collected for an LDH leakage assay. The LDH leakage rates of HepG2 and L02 cells with TSN exposure were significantly increased in a concentration-dependent manner (Figure 1C,D). In addition, observation of the cell morphology was used to intuitively reflect the damage of TSN to HepG2 cells. With the increase in concentration and time of TSN exposure, the viable cell counting of HepG2 cells decreased, and the cell morphology changed, mainly shrinking and rounding, compared with the control group (Figure 1E). These results showed that TSN had obvious cellular toxicity on HepG2 and L02 cells.

### 2.2. TSN Inhibits Autophagic Flux in HepG2 Cells

**Figure 2:**
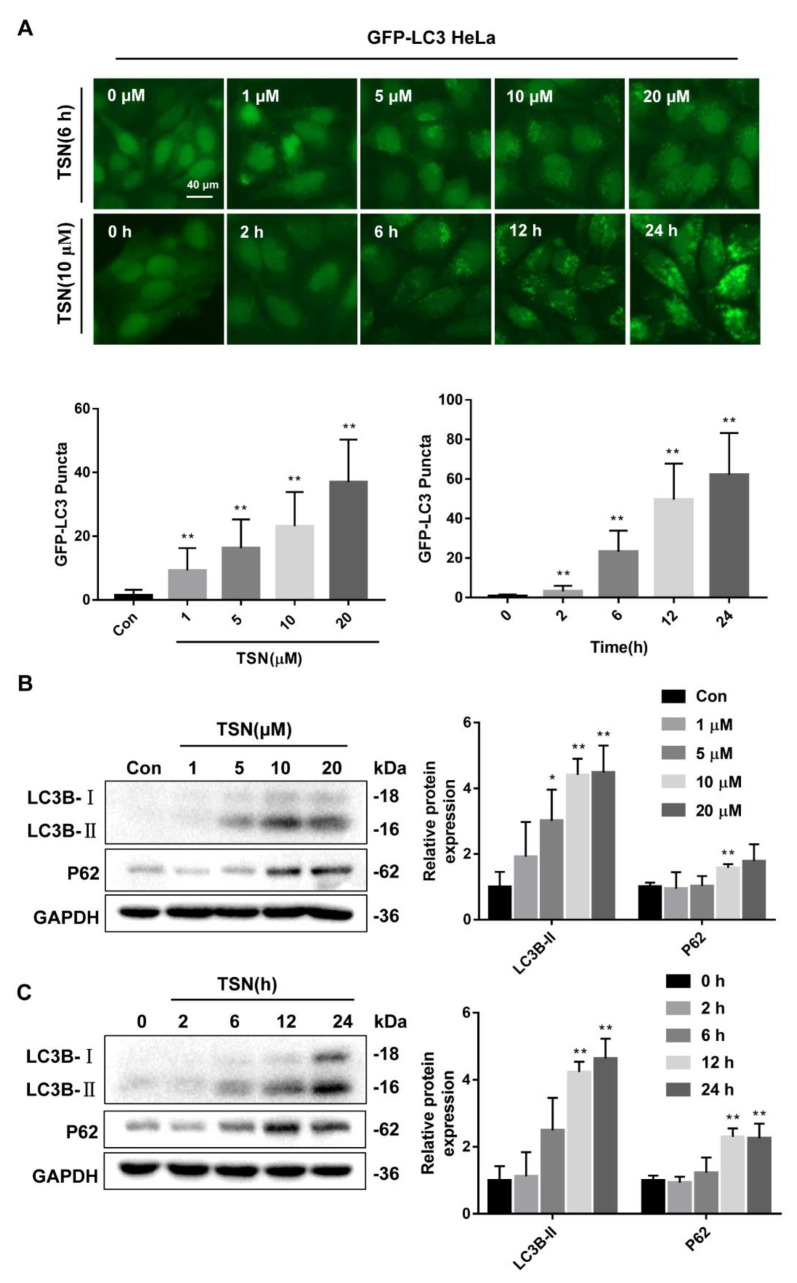

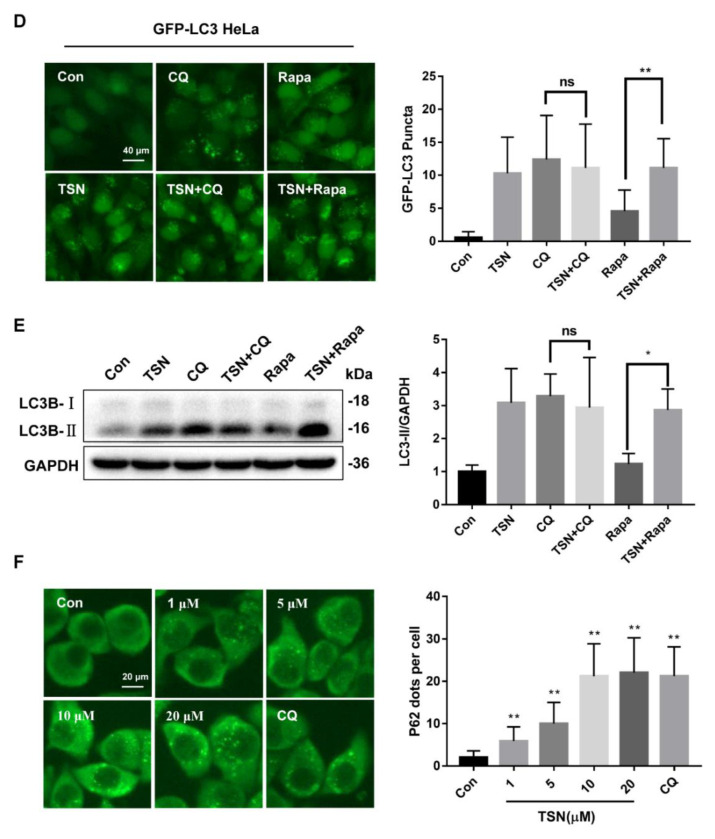
TSN inhibited autophagic flux in HepG2 cells. (**A**) HeLa cells expressing GFP-LC3 were treated with TSN for 6 h at different concentrations (1, 5, 10, 20 µM) or were treated with TSN (10 µM) over a certain time course (0, 2, 6, 12, 24 h). The distribution of GFP-LC3 was examined, and the number of GFP-LC3 puncta was quantified. Scale bar: 40 µm. (**B**) HepG2 cells treated with TSN for 6 h at different concentrations (1, 5, 10, 20 µM) were analyzed by Western blotting for the protein levels of LC3B and P62. The ratios of LC3B-II/GAPDH and P62/GAPDH were calculated using ImageJ software. (**C**) HepG2 cells treated with TSN (10 µM) over a certain time course (0, 2, 6, 12, 24 h) were analyzed by Western blotting for the protein levels of LC3B-II and P62. The ratios of LC3B-II/GAPDH and P62/GAPDH were calculated using ImageJ software. (**D**) HeLa cells expressing GFP-LC3 were treated by TSN (10 µM) with or without chloroquine (CQ, 40 µM) or rapamycin (Rapa, 1 µM) for 6 h. The distribution of GFP-LC3 was examined, and the number of GFP-LC3 puncta was quantified. Scale bar: 40 µm. (**E**) HepG2 cells were treated by TSN (10 µM) with or without CQ (40 µM) or Rapa (1 µM) for 6 h. The endogenous LC3B was analyzed by Western blotting, and the ratio of LC3B-II/GAPDH was calculated using ImageJ software. (**F**) HepG2 cells were treated with TSN at different concentrations (1, 5 10, 20 µM) or CQ (40 µM) for 6 h. Cellular immunofluorescence staining was used to detect P62, and the number of P62 puncta was quantified. Scale bar: 20 µm. Data are presented as the mean ± SD of three independent experiments. * *p* < 0.05, ** *p* < 0.01 versus control; ns, not significant.

To determine the effect of TSN on autophagy, HeLa cells stably expressing green fluorescent protein (GFP)-LC3 were employed. GFP-LC3 HeLa and HepG2 cells were treated with TSN for 6 h at different concentrations (1, 5, 10, 20 µM) or treated with TSN (10 µM) over a certain time course (0, 2, 6, 12, 24 h). TSN induced the accumulation of GFP-LC3 puncta in concentration- and time-dependent manners (Figure 2A). Then, the expression levels of LC3B-II and P62 were analyzed by Western blotting. The results showed that TSN caused the concentration- and time-dependent upregulation of LC3B-II and P62 in HepG2 cells (Figure 2B,C). Moreover, the mRNA levels of *ATG4B* and *ATG7* decreased, and the mRNA level of *ATG12* increased slightly; the mRNA levels of *LC3B* and *P62* increased significantly, while the mRNA levels of *Beclin1*, *ATG5*, and *ATG10* did not change under TSN treatment (Supplementary Figure S1). The results showed that TSN generally had no obvious effect on the mRNA level of the genes related to early autophagy. To further confirm the effect of TSN on autophagic flux, autophagy inhibitor CQ and activator Rapa were used to measure the turnover of LC3-II. GFP-LC3 HeLa and HepG2 cells were treated by TSN (10 µM) with or without CQ (40 µM) or rapamycin (Rapa) (1 µM) for 6 h. TSN, CQ, and Rapa could all cause the increase in GFP-LC3 puncta. However, TSN treatment did not further increase the CQ-induced accumulation of GFP-LC3 puncta, while it remarkably enhanced the Rapa-induced accumulation of GFP-LC3 puncta (Figure 2D). Similarly, the relative expression levels of LC3B-II were upregulated in the TSN-, CQ-, and Rapa-alone groups compared with the control group for HepG2 cells. There was no difference in the protein level of LC3B-II between the CQ-alone group and the TSN-plus-CQ group. Nevertheless, TSN further raised Rapa-induced upregulation of the LC3B-II protein level (Figure 2E). In addition, cellular immunofluorescence staining showed that TSN increased P62 puncta in a concentration-dependent manner (Figure 2F). The above results indicate that TSN is an effective inhibitor of autophagic flux.

### 2.3. TSN Increases the Accumulation of Autolysosomes in HepG2 Cells and L02 Cells

**Figure 3:**
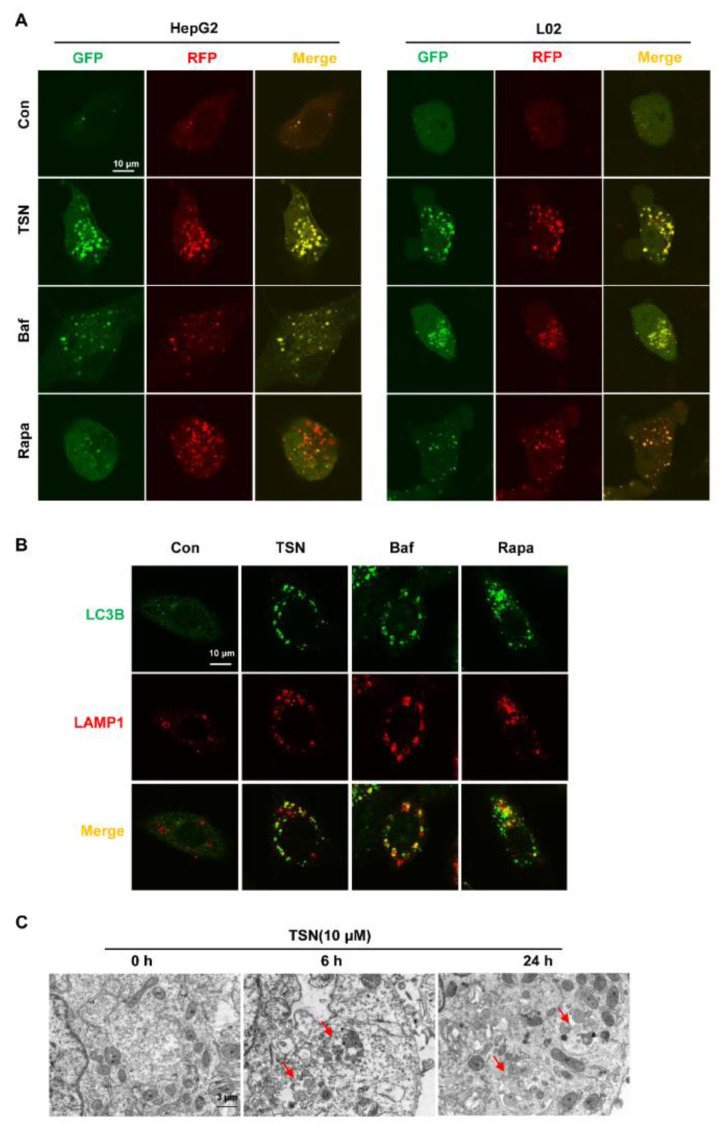
TSN increased the accumulation of autolysosomes in HepG2 cells and L02 cells. (**A**) HepG2 cells and L02 cells transfected with an RFP–GFP–LC3 tandem sensor were treated with TSN (10 µM), bafilomycin A1 (Baf, 0.5 µM), or Rapa (1 µM) for 6 h. The colocalization of GFP and RFP puncta was imaged using confocal microscopy. Scale bar: 10 µm. (**B**) HepG2 cells were treated with TSN (10 µM), Baf (0.5 µM), or Rapa (1 µM) for 6 h, and then cellular immunofluorescence staining was used to detect the colocalization of LC3B and LAMP1. Scale bar: 10 µm. (**C**) HepG2 cells were treated with TSN (10 µM) for a certain time course (0, 6, 24 h), and then transmission electron microscopy (TEM) was used to detect the ultrastructure of HepG2 cells. Red arrows indicate autolysosome structures. Scale bar: 3 µm. Data are presented as the representative figures of three independent experiments.

To observe the changes of autophagic flux caused by TSN, a red fluorescent protein (RFP)–GFP–LC3 tandem sensor was used to assess the autophagy system. During autophagy, the acidic environment of lysosomes can lead to a decrease in GFP fluorescence because it makes the latter unstable, whereas more RFP fluorescence puncta can be observed because RFP possesses acid-resisting properties [32]. HepG2 and L02 cells transfected with an RFP–GFP–LC3 tandem sensor were treated with TSN (10 µM), autophagy inhibitor Bafilomycin A1 (Baf), or Rapa (1 µM) for 6 h. Compared with the control group, the Rapa group significantly increased the RFP fluorescence puncta in HepG2 and L02 cells. However, similar to the Baf group, TSN treatment increased both the RFP and GFP fluorescent puncta that produced colocalization (puncta formation with yellow overlay) (Figure 3A), indicating TSN acted on the middle or late stages of autophagy. In these stages, autophagosomes fuse with lysosomes to generate autolysosomes, and then the contents are degraded. The effect of TSN on the fusion of autophagosomes and lysosomes was detected by determining the localization of the lysosome membrane marker LAMP1 and the autophagosome marker LC3B. Similar to the TSN group, autophagosomes could not be degraded after fusion with lysosomes, so the RFP and GFP puncta co-located to form yellow puncta in the HepG2 cells in the Baf group by inhibiting the degradation of lysosomes. Conversely, relatively fewer yellow puncta were observed in Rapa-treated cells (Figure 3B). These results suggest that TSN did not inhibit the fusion of autophagosomes and lysosomes. In addition, HepG2 cells were treated with TSN (10 µM) over a certain time course (0, 6, 24 h), and then the ultrastructure of HepG2 cells was detected with transmission electron microscopy (TEM). Obvious autolysosomes were observed in the TSN-treated HepG2 cells, indicating that TSN could cause the accumulation of autolysosomes (Figure 3C).

### 2.4. TSN Induces Lysosome Dysfunction in Vitro

**Figure 4:**
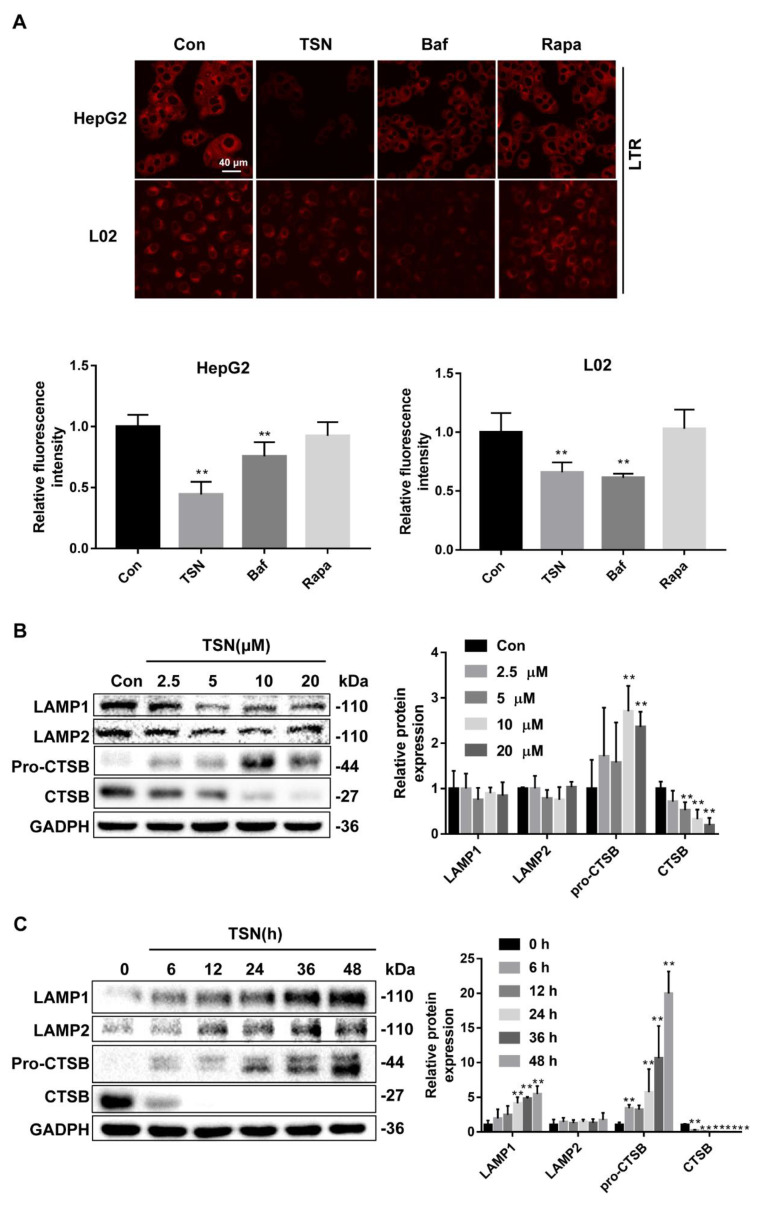

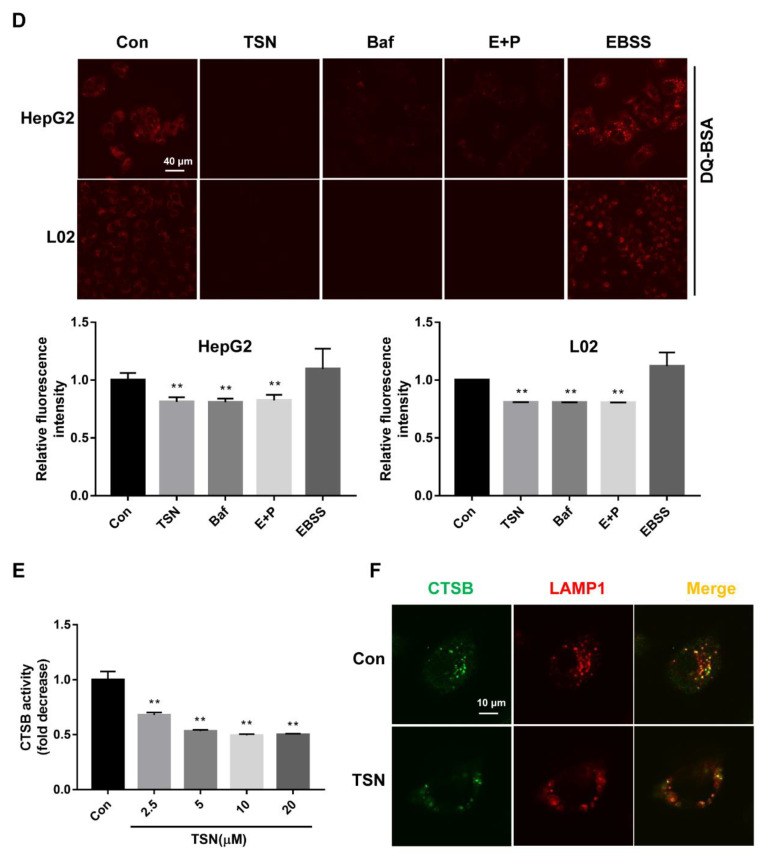
TSN induced lysosome dysfunction in HepG2 cells and L02 cells. (**A**) HepG2 cells and L02 cells were treated with TSN (10 µM), Baf (0.5 µM), or Rapa (1 µM) for 6 h, then lysosomal acidity was monitored by LTR staining, and the relative LTR fluorescent intensity was quantified. Scale bar: 40 µm. (**B**) HepG2 cells treated with TSN for 6 h at different concentrations (2.5, 5 10, 20 µM) were analyzed by Western blotting for the protein levels of LAMP1, LAMP2, and CTSB. The ratios of LAMP1/GAPDH, LAMP2/GAPDH, and CTSB/GAPDH were calculated using ImageJ software. (**C**) HepG2 cells treated with TSN (10 µM) over a certain time course (0, 6, 12, 24, 36, 48 h) were analyzed by Western blotting for the protein levels of LAMP1, LAMP2, and CTSB. The ratios of LAMP1/GAPDH, LAMP2/GAPDH, and CTSB/GAPDH were calculated using ImageJ software. (**D**) HepG2 cells and L02 cells were firstly incubated with 10 µg/mL DQ-BSA in EBSS medium for 1.5 h and then treated with TSN (10 µM), Baf (0.5 µM), E64D (25 µM) plus pepstatin A (50 µM) (E + P), and EBSS medium for another 6 h. The relative DQ-BSA fluorescent intensity was quantified. Scale bar: 40 µm. (**E**) HepG2 cells were treated with TSN for 6 h at different concentrations (2.5, 5, 10, 20 µM), and then CTSB activity was assayed using a CTSB Activity Assay Kit (Fluorometric). (**F**) HepG2 cells were treated with or without TSN (10 µM) for 6 h, and then cellular immunofluorescence staining was used to detect the colocalization of CTSB and LAMP1. Scale bar: 10 µm. Data are presented as the mean ± SD of three independent experiments. ** *p* < 0.01 versus control.

Given the important role of lysosomes in the late stage of autophagy, we assessed the lysosomal acidity, expression levels of membrane associated proteins, and hydrolase CTSB, as well as the lysosomal degradation function, in the TSN group. The lysosomal acidity was monitored by LysoTracker Red (LTR) staining in HepG2 and L02 cells treated with TSN (10 µM), Baf (0.5 µM), or Rapa (1 µM) for 6 h. Compared with the control group, TSN and Baf inhibited the acidic environment of lysosomes to varying degrees, while Rapa had no effect on the acidity of lysosomes (Figure 4A). Then, HepG2 cells treated with TSN for 6 h at various concentrations (2.5, 5, 10, 20 µM) or at 10 µM over a certain time course (0, 6, 12, 24, 36, 48 h) were analyzed by Western blotting for the protein levels of LAMP1, lysosomal membrane proteins 2 (LAMP2), and CTSB. TSN increased LAMP1 expression in a time-dependent manner, while the level of LAMP2 protein did not change. It should be noted that TSN significantly reduced the expression of CTSB in both concentration-dependent and time-dependent manners (Figure 4B,C). Next, Dye Quenched-Bovine Serum Albumin (DQ-BSA) was used to detect the effect of TSN on lysosomal degradation. Similar to normal lysosomal conditions, DQ-BSA in the control group could be degraded, then releasing red fluorescence. Compared with the control group, TSN, Baf, and E64D plus pepstatin A (two lysosomal hydrolase inhibitors) treatment significantly inhibited the degradation ability of lysosomes, which was different from the result of starvation treatment (Earle’s balanced salt solution, EBSS group) (Figure 4D). Moreover, the enzyme activity of CTSB decreased in a concentration-dependent manner with TSN treatment (Figure 4E). To determine whether TSN caused lysosome membrane permeabilization, the colocalization of CTSB and LAMP1 was evaluated. Similar to the control group, TSN did not affect the colocalization of CTSB and LAMP1, indicating that TSN did not change the integrity of the lysosomal membrane (Figure 4F). Taken together, the above results suggest that TSN causes lysosomal dysfunction.

### 2.5. TSN Blocks Autophagic Flux by Inhibiting TFEB in Vitro

**Figure 5:**
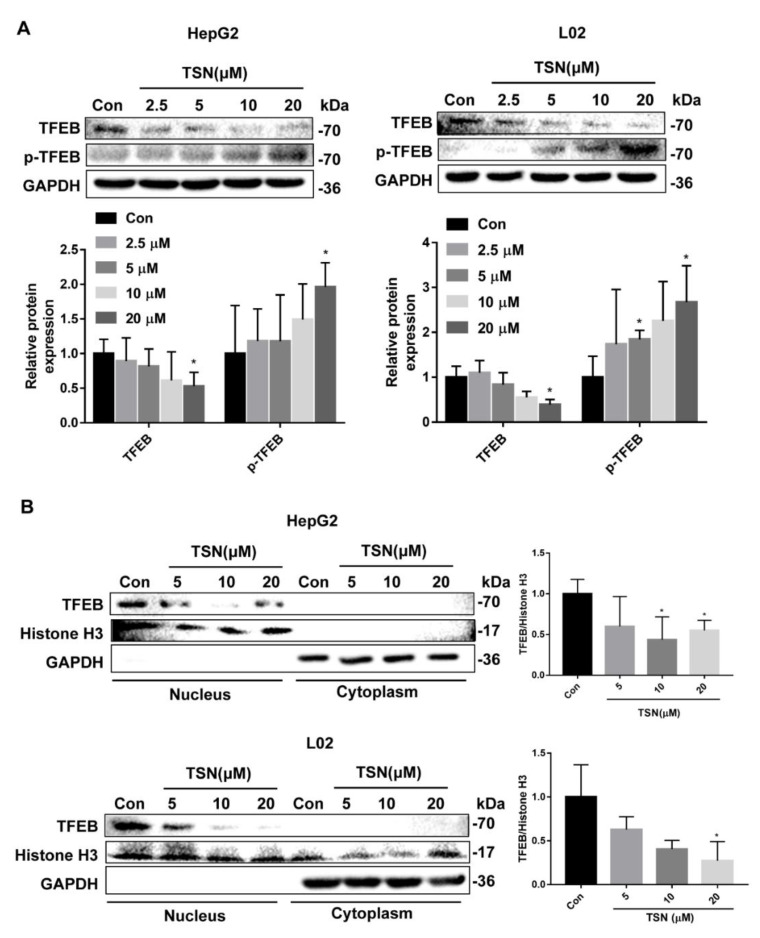

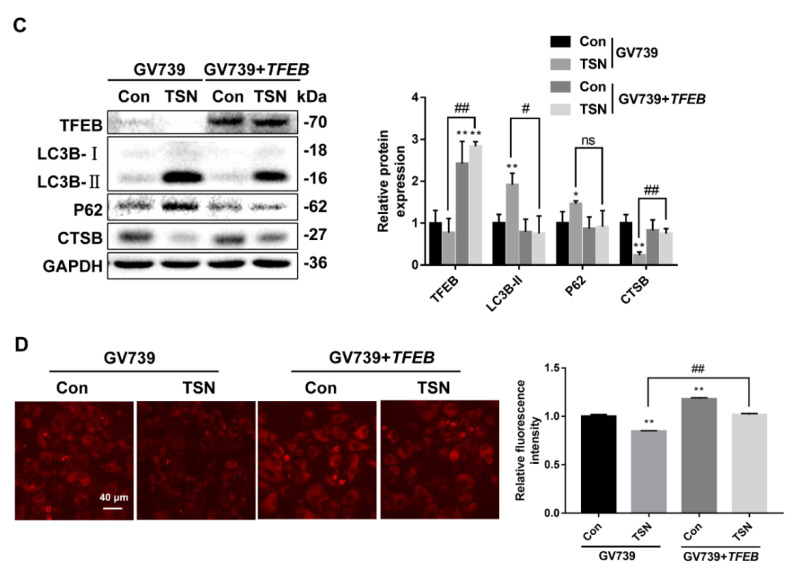
TSN blocked autophagic flux by inhibiting TFEB in vitro. (**A**) HepG2 cells and L02 cells treated with TSN for 6 h at different concentrations (2.5, 5 10, 20 µM) were analyzed by Western blotting for the protein levels of TFEB and p-TFEB (Ser211). (**B**) HepG2 cells and L02 cells were treated with TSN for 6 h at different concentrations (2.5, 5 10, 20 µM). The expression levels of TFEB in nuclear and cytosolic fractions were determined using Western blotting. (**C**,**D**) HepG2 cells were transfected with GV739 plasmid or *TFEB*-GV739 plasmid and then were treated by TSN (10 µM) for 6 h. The protein levels of TFEB, LC3B, P62, and CTSB were determined by Western blotting (**C**). Lysosomal acidity was monitored by LTR staining, and the relative LTR fluorescent intensity was quantified (**D**). Scale bar: 40 µm. Data are presented as the mean ± SD of three independent experiments. Relative protein expression levels were calculated using ImageJ software. * *p* < 0.05, ** *p* < 0.01 versus vehicle control. ns, not significant. ^#^ *p* < 0.05, ^##^ *p* < 0.01 versus TSN group in cells transfected with empty plasmid GV739.

TFEB is a crucial transcription factor that controls autophagy and lysosomal biogenesis. To explore whether TSN exerted autophagic flux blockade and liver injury effects through TFEB, the expression level of TFEB was detected. TSN treatment induced the downregulation of active TFEB and upregulation of inactive p-TFEB (Ser211) in a concentration-dependent manner in HepG2 and L02 cells (Figure 5A). Since TFEB mainly functions by translocating to the nucleus, the expression level of nuclear TFEB was detected. TFEB expression in the nucleus also decreased under TSN treatment in a concentration-dependent manner in HepG2 and L02 cells (Figure 5B). Then, HepG2 cells were transfected with GV739 plasmid (negative control) or *TFEB*-GV739 plasmid and were treated by TSN (10 µM) for 6 h. The results showed that overexpression of TFEB significantly reversed the upregulation of LC3B-II and P62 induced by TSN and upregulated the expression level of CTSB (Figure 5C). In addition, overexpression of TFEB could restore the lysosomal pH disturbed by TSN (Figure 5D). The above results suggested that TSN blocked autophagic flux by inhibiting TFEB.

### 2.6. Activation of Autophagy Alleviates TSN-Induced Cell Damage

**Figure 6:**
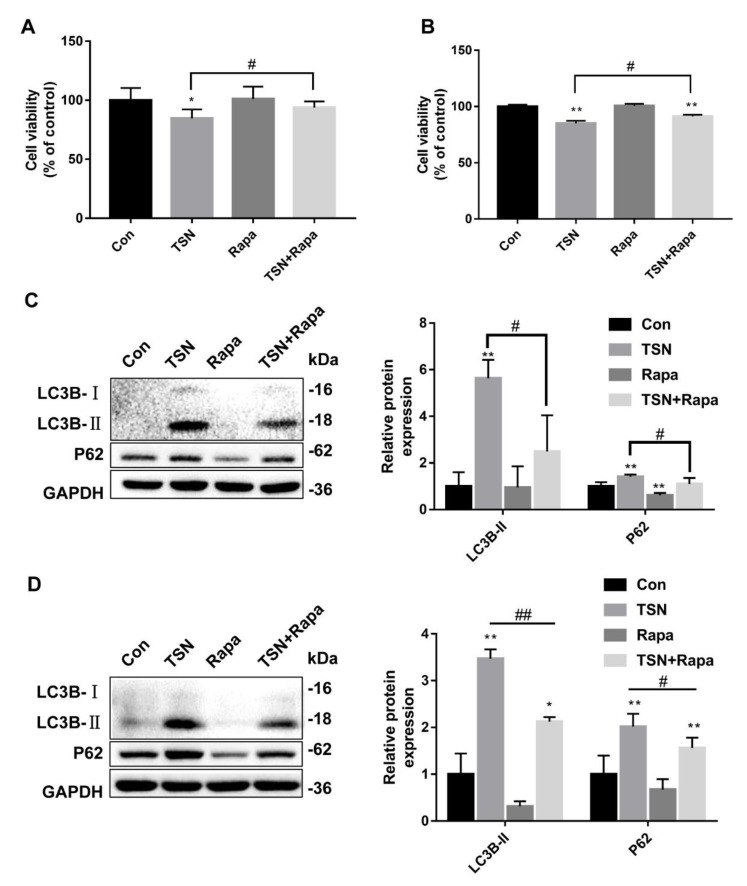
Activation of autophagy alleviated TSN-induced cell damage. HepG2 and L02 cells were treated by TSN (10 µM) with or without Rapa (1 µM) for 24 h. (**A**) (HepG2) and (**B**) (L02) cell viability was determined using a CCK-8 assay kit. (**C**) (HepG2) and (**D**) (L02) protein levels for LC3B and P62 were analyzed by Western blotting, and the ratios of LC3B-II/GAPDH and P62/GAPDH were calculated using ImageJ software. Data are presented as the mean ± SD of three independent experiments. * *p* < 0.05, ** *p* < 0.01 versus control; ^#^ *p* < 0.05, ^##^ *p* < 0.01 versus TSN group.

To explore the relationship between the TSN-induced inhibition of autophagic flux and hepatotoxicity, the CCK-8 assay was used to assess the effect of autophagy activation on cell viability in HepG2 and L02 cells incubated with TSN. HepG2 and L02 cells were subjected to TSN (10 µM) treatment, with or without Rapa (1 µM), for 24 h. In comparison to the control group, cell viability was inhibited in the TSN-alone treatment group but increased after co-treatment with Rapa (Figure 6A,B). Moreover, the expression levels of LC3B-II increased in the TSN-alone treatment group in comparison to the control yet decreased after co-treatment with Rapa, similar to the results for P62 (Figure 6C,D). The results showed that autophagy activation alleviated TSN-induced cell damage.

### 2.7. TSN Induces Liver Injury in Balb/c Mice

**Figure 7:**
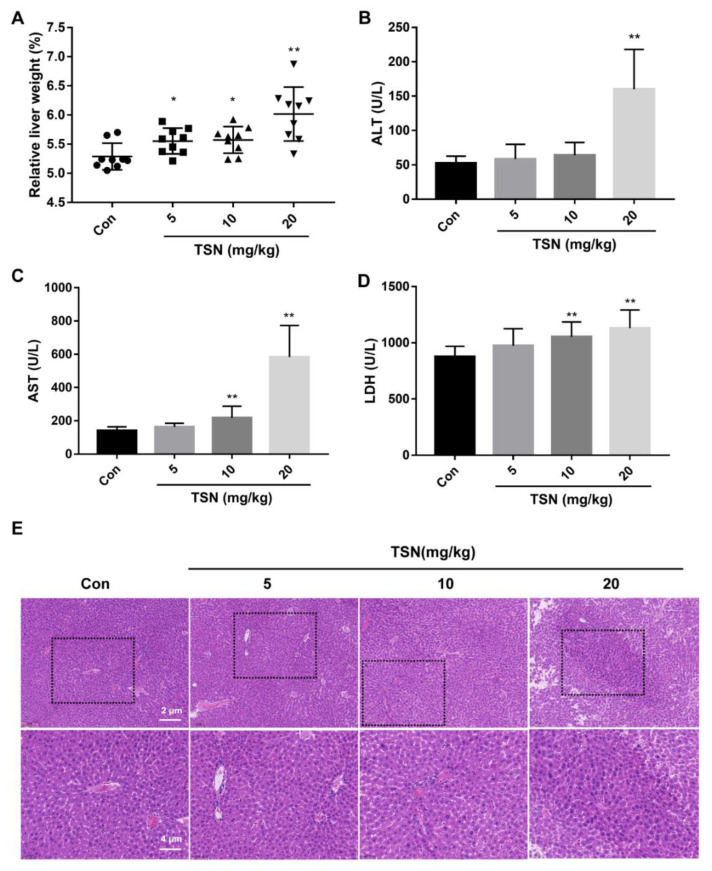
TSN induced liver injury in Balb/c mice. Balb/c mice were intraperitoneally administered TSN at different doses (5, 10, 20 mg/kg) or by vehicle for 24 h. Serum and liver tissue were harvested after TSN treatment. (**A**) Liver index were calculated from vehicle and TSN-treated groups. The effects of TSN on the levels of ALT (**B**), AST (**C**), and LDH (**D**) in serum were measured. (**E**) The effects of TSN on the histological changes in mice liver tissues are indicated by H&E staining. Scale bar: 2 µm (above) and 4 µm (below). Data are presented as the mean ± SD * *p* < 0.05, ** *p* < 0.01 versus vehicle, *n* = 9.

The hepatotoxicity of TSN in vivo was investigated. Balb/c mice were intraperitoneally administered TSN at different doses (5, 10, 20 mg/kg) or by vehicle for 24 h. Compared with the vehicle group, the relative liver weight (liver weight to body weight ratio) in the TSN-administration groups increased in a dose-dependent manner (Figure 7A). Serum ALT, AST, and LDH levels were elevated in TSN-administration mice (Figure 7B–D). The results of a liver histological evaluation further confirmed the liver injury caused by TSN. In the vehicle group, the structure of hepatic sinusoid was clear, the structure of hepatocytes was intact, and the arrangement of hepatocytes was ordered. However, especially in the high-dose TSN group, hepatocyte necrosis, nuclear pyknosis, and the nuclear disappearance of some hepatocytes were observed (Figure 7E). The above results showed that TSN induced acute liver injury in mice.

### 2.8. TSN Inhibits Autophagic Flux and Induces Lysosome Dysfunction in Balb/c Mice

**Figure 8:**
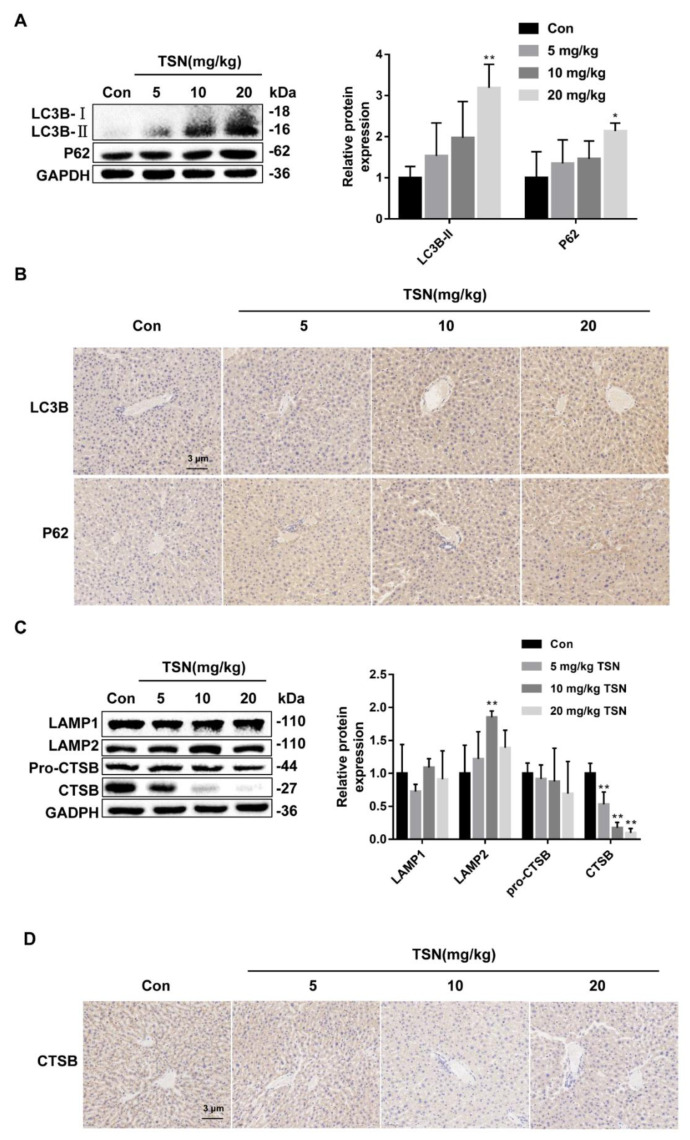

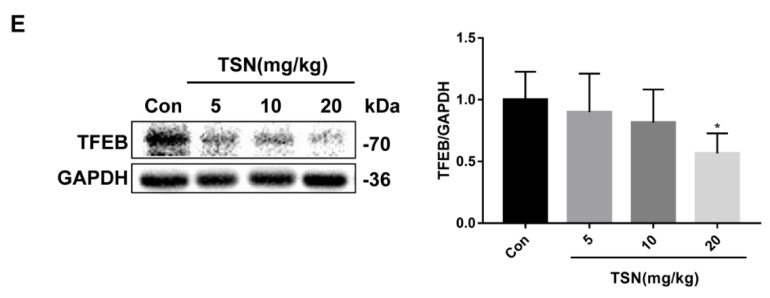
TSN inhibited autophagic flux and induced lysosome dysfunction in Balb/c mice. Balb/c mice were intraperitoneally administered TSN at different doses (5, 10, 20 mg/kg) or by vehicle for 24 h. Serum and liver tissue were harvested after TSN treatment. (**A**) The protein levels of LC3B and P62 were determined by Western blotting, and the ratios of LC3B-II/GAPDH and P62/GAPDH were calculated using ImageJ software. (**B**) Representative results of IHC staining for LC3B and P62. Scale bar: 3 µm. (**C**) The protein levels of LAMP1, LAMP2, and CTSB were determined by Western blotting, and the ratios of LAMP1/GAPDH, LAMP2/GAPDH, and CTSB/GAPDH were calculated using ImageJ software. (**D**) Representative results of IHC staining for CTSB. Scale bar: 3 µm. (**E**) The protein level of TFEB was determined by Western blotting. Data are presented as the mean ± SD * *p* < 0.05, ** *p* < 0.01 versus vehicle, *n* = 9.

To further verify the effect of TSN on autophagic flux and lysosomal function in vivo, the expression levels of autophagy-related proteins and lysosomal-related proteins were detected. Western blotting showed that the expression of LC3B-II and P62 in the TSN-administration groups were upregulated in a dose-dependent manner (Figure 8A). The upregulation of LC3B-II and P62 induced by TSN in vivo was further verified by immunohistochemistry (IHC) staining (Figure 8B). In addition, TSN downregulated the expression of CTSB in mice livers, which was consistent with the results in vitro. The expression level of LAMP1 was not affected by TSN administration, while the expression level of LAMP2 was up-regulated in a dose-dependent manner (Figure 8C). Interestingly, the TSN-induced downregulation of CTSB in vivo was further verified by IHC staining (Figure 8D). A dose-dependent downregulation of TFEB expression by TSN was also observed in mouse liver tissue (Figure 8E). Based on these data, TSN inhibited autophagic flux and induced lysosome dysfunction in vivo.

## 3. Discussion

*Meliae Toosendan* is one of the widely reported hepatotoxic traditional Chinese medicines, and its main toxic ingredient is TSN. At present, few studies mention that autophagy may be involved in TSN-induced hepatotoxicity [26]. However, the specific effect of TSN on autophagy in hepatocytes, especially on functional autophagic flux, is unknown. On these grounds, this study explored the effect of TSN on hepatocyte autophagy and the role of autophagy in TSN hepatotoxicity. The data showed that the proliferation of HepG2 and L02 cells with TSN treatment was inhibited, the release rate of LDH increased, and the morphology and number of cells changed significantly, which demonstrated that TSN had obvious hepatotoxicity. TSN induced the accumulation of GFP-LC3 puncta and the upregulation of LC3B-II and P62 protein expressions in time-dependent and concentration-dependent manners. The combination of autophagy-inhibitor CQ or autophagy-inducer Rapa with TSN and the increase in autolysosomes’ number in HepG2 cells suggested that TSN inhibited the autophagic flux. Further, TSN restrained lysosomal acidic environment and degradation ability and inhibited the activity and expression level of lysosomal hydrolase CTSB. Rapa combined with TSN alleviated the decrease in cell viability caused by TSN, indicating that TSN-induced blockade of autophagic flux was one important factor for its hepatotoxicity. TSN had a significant inhibitory effect on the expression of TFEB, a master transcription factor for lysosomal biogenesis and functions, while overexpression of TFEB reversed the blockade of autophagic flux and restored lysosomal pH, indicating that TSN-induced inhibition of autophagic flux is TFEB-dependent. Overall, our study demonstrated that TSN induced obvious hepatotoxicity through TFEB-lysosome-mediated autophagic flux blockade.

Human hepatoblastoma HepG2 cell is a prominent cell model for toxic-induced hepatotoxicity evaluations in vitro [33]. Among the hepatic cell lines, HepG2 cells first showed the key characteristics of hepatocytes and have retained most of the metabolic functions of normal hepatocytes [34]. So HepG2 is the main cell line used in this study, and it is sensitive to the cytotoxicity of TSN. However, studies have shown that the expression level of cytochromes (CYP) genes (mainly CYP2B6, CYP3A4) in HepG2 is lower than that in normal hepatocytes [35]. HepG2 cells cannot fully represent the phenotype of hepatocytes, as it is not accurate enough to evaluate many hepatotoxic compounds by using HepG2 cell lines alone. Therefore, a second cell line model, human normal liver cell line L02, was employed to further evaluate the hepatotoxicity of TSN and explore its toxic mechanism. Moreover, HeLa (cervical cancer cell) stably expressing GFP-LC3 was used for the tool cells to detect the impact of TSN on LC3. After finding that TSN induced the LC3 puncta accumulation in GFP–LC3 HeLa tool cells, we then detected the protein expression level of LC3B on HepG2 cells to verify the accumulation effect of TSN on LC3 in liver cells. The hepatotoxicity of TSN and its inhibition effects on autophagic flux and lysosomes were also confirmed in the mouse model. Altogether, this study convincingly shows that TSN induced hepatotoxicity through TFEB-lysosome-mediated autophagic flux blockade.

Autophagy is the main degradation form of damaged organelles and macromolecular proteins in eukaryotic cells, which is of great significance for maintaining cellular homeostasis. In recent years, rising attention has been paid to the relationship between autophagy and DILI. For example, the hepatotoxic mechanism of acetaminophen, the main cause of acute liver failure, has been widely studied. It has been reported that excessive acetaminophen is metabolized to produce the reactive metabolite N-acetyl-p-phenylquinone imine (NAPQI), which combines with GSH and exhausts GSH, leading to the binding of NAPQI with intracellular protein to cause mitochondrial dysfunction, liver cell necrosis, liver damage, and possible liver failure [22,36]. Acetaminophen could activate or inhibit autophagy, which may be dependent on different treatment conditions [37,38]. Interestingly, whether autophagy is activated or inhibited by acetaminophen, the activation of autophagy could prevent acetaminophen-induced liver injury [37,38]. The protective effect of autophagy may be due to the selective removal of acetaminophen adducts or damaged mitochondria [22]. In this study, TSN inhibited TFEB-lysosome-dependent autophagic flux. Similar to acetaminophen, activated autophagy (by rapamycin or overexpression of TFEB) could restore TSN-inhibited autophagic flux and reduce hepatotoxicity. This study further enhanced our understanding of the molecular mechanism of autophagy-mediated DILI protection, and the activation of autophagy provides a better option for DILI patients.

Lysosomes, as the degradation center of cells, participate in the fusion of autophagosomes and lysosomes and the degradation of the contents. However, the effect of TSN on lysosomes was unknown. Our data showed that TSN did not inhibit the colocalization of LC3B and LAMP1, indicating that TSN did not affect autophagic flux by inhibiting autophagosome–lysosome fusion. TSN also significantly inhibited lysosomal acidity, the expression and activity of protease CTSB, and degradation ability. The low pH value in lysosomes provides an acidic environment for a large number of hydrolases with a normal expression level and activity that are the key factors for performing the degradation function of lysosomes. CTSB is a lysosomal cysteine protease that regulates the occurrence and development of tumor, liver fibrosis, obesity, Alzheimer’s disease, and other diseases [39,40,41,42]. Interestingly, our data showed that TSN caused a significant decrease in CTSB protein expression both in vivo and in vitro and in CTSB enzyme activity in vitro. In general, TSN showed a multifaceted and significant lysosomal inhibitory capacity, leading to the inhibition of autophagic flux.

TFEB is a key transcription factor controlling autophagy and lysosomal biogenesis. TFEB-dependent autophagy activation has been proven to play a protective role in renal injury induced by multiple drugs [30,43,44]. So whether TFEB could play a role in TSN-induced hepatotoxicity has aroused our research interest. This study found that the total and nuclear protein levels of TFEB in hepatocytes were significantly downregulated by TSN. Overexpression of TFEB could reverse the accumulation of LC3B-II and P62 proteins caused by TSN, upregulate the protein expression of target gene CTSB, and restore the normal lysosomal pH, suggesting that TFEB-dependent dysfunction of autophagy is involved in TSN-induced hepatotoxicity; thus, targeting TFEB to regulate autophagy may become a new therapeutic strategy for TSN-induced hepatotoxicity.

## 4. Material and Methods

### 4.1. Materials

TSN (purity > 98%, MB6571) was purchased from Meilunbio Company (Dalian, China). Chloroquine (CQ, purity 99.50%, HY-17589A), rapamycin (Rapa, purity 99.81%, HY-10219), Bafilomycin A1 (Baf, purity > 99.0%, HY-100558), E64D (purity 99.55%, HY-100229), and pepstatin A (purity 98.28%, HY-P0018) were purchased from MedChemExpress (Monmouth Junction, NJ, USA). Anti-LC3B (18725-1-AP), anti-P62 (18420-1-AP), anti-LAMP2 (66201-1-Ig), and anti-GAPDH (60004-1-Ig) primary antibodies were purchased from Proteintech (Rosemont, IL, USA). Anti-LAMP1 (#15665), anti-CTSB (#31718), anti-TFEB (#37785), anti-p-TFEB(Ser211) (#37681), and anti-Histone H3(#4499) primary antibodies were purchased from Cell Signaling Technology (Billerica, CA, USA).

### 4.2. Cell Culture

Human hepatoblastoma HepG2 cells were purchased from the Chinese Academy of Science cell bank (Shanghai, China). The human hepatic L02 cells and GFP-LC3 HeLa cells [45,46] were kindly gifted by Min Li (School of Pharmaceutical Sciences, Sun Yat-sen University, Guangdong, China). HepG2 cells and GFP-LC3 HeLa cells were cultured in Dulbecco’s Modified Eagle’s Medium (DMEM) high glucose medium (Biological Industries, Kibbutz Beit Haemek, Israel), with 10% fetal bovine serum (Gibco, Grand Island, NY, USA). L02 cells were cultured in RPMI-1640 medium (Biological Industries, USA), with 10% fetal bovine serum (Biological Industries, USA). All of the media were supplemented with 100 U/mL penicillin and 100 µg/mL streptomycin (Gibco, Grand Island, NY, USA). All of the cells were incubated at 37 °C in a humidified atmosphere with 5% CO_2_.

### 4.3. Cell Transfection

HepG2 cells at 70% confluency were transiently transfected with *TFEB*-GV739 plasmid or GV739 vector purchased from GeneChem Co., Ltd. (Shanghai, China) using Lipofectamine™ 3000 (Invitrogen, Carlsbad, CA, USA). Following transfected for 48 h, cells were treated by TSN (10 µM) for 6 h to perform the assigned assays.

### 4.4. Cell Viability Assay

Cell viability was determined by CCK-8(Bimake, Houston, TX, USA), in accordance with the instructions of the manufacturer. HepG2 and L02 cells were seeded in 96-well cell culture plates at a density of 5 × 10^4^ cells/mL and incubated for 12 h. The cells were treated with different concentrations of TSN for 24 h. At the end of the incubation, 10 µL CCK-8 solution was added to each well and incubated at 37 °C for 1 h. The absorbance was detected at 450 nm by using a microplate reader (Thermo Multiskan Sky, Waltham, MA, USA), and then the cell viability was calculated.

### 4.5. LDH Leakage Assay

HepG2 and L02 cells were seeded in 96-well cell culture plates at a density of 5 × 10^4^ cells/mL and incubated for 12 h. The cells were treated with different concentrations of TSN for 24 h. Cell culture supernatants were collected, and the level of LDH leakage was measured by a microplate reader (Thermo Multiskan Sky, USA), in accordance with the instructions of the manufacturer, using the LDH Assay Kit (Abcam, Cambridge, MA, USA).

### 4.6. Analysis of Cell Morphology

HepG2 cCells were seeded in 24-well cell culture plates at a density of 2 × 10^4^ cells/mL and incubated for 12 h. The cells were treated with different concentrations of TSN (2.5, 5, 10 µM) for different time durations (0, 6, 24, 48 h). After treatment, the changes in cell morphology were observed under phase-contrast microscopy (Olympus, Tokyo, Japan).

### 4.7. Western Blot Analysis

Total protein fractions of HepG2 cells, L02 cells, and liver tissue were obtained by RIPA lysis buffer (Beyotime, Shanghai, China) containing 2% protease inhibitor cocktail and 1% phosphatase inhibitor. Nuclear and cytosolic proteins were prepared by a Nuclear and Cytoplasmic Protein Extraction Kit (Beyotime, Shanghai, China). Samples containing 30 µg of protein were separated by SDS-PAGE. Then, the proteins were transferred to polyvinylidene difluoride (PVDF) membranes (Merck Millipore, Billerica, MA, USA). The membranes were blocked with 5% nonfat milk at room temperature for 1 h and then incubated with primary antibodies overnight at 4 °C. After washing with TBST (20 mM Tris, 150 mM NaCl, 2.7 mM KCl, 0.1% Tween 20, pH 7.4) solution, the membranes were incubated with HRP-conjugated secondary antibody at room temperature for 1 h. After washing with TBST solution, an electrochemiluminescence (ECL) kit (Thermo Fisher Scientific, Waltham, MA, USA) was used to develop the membranes, in accordance with the protocol of the manufacturer. Each band was visualized by a chemiluminescence detection system (BioRad Laboratories, Hercules, CA, USA). Gray values of protein bands were analyzed by ImageJ Software.

### 4.8. Quantitative Real-Time PCR Analysis

Total RNA was extracted from cells using RNA simple total kit (Tiangen, Beijing, China). After reverse transcription using the M-MLV reverse transcriptase kit (Accurate Biology, Changsha, Hunan, China), complementary DNA (cDNA) was prepared to quantitative PCR with SYBR Green (Accurate Biology, Changsha, Hunan, China), in accordance with the instructions of the manufacturer. The primer sequences (Sangon Biotech, Shanghai, China) for the target genes are shown in Supplementary Table S1. Subsequent PCR amplification was conducted on a LightCycler 480 II system (Roche Diagnostics, Basel, Switzerland). The relative mRNA expression was analyzed by using the 2^−ΔΔCt^ method, and GAPDH was used as the internal control.

### 4.9. Immunofluorescence Staining

HepG2 cells were seeded in confocal dishes at a density of 4 × 10^4^ cells/mL and incubated for 12 h. After treatment with the specified drugs for 6 h, HepG2 cells were fixed with 4% paraformaldehyde (PFA) for 20 min, permeated with immunostaining permeabilization buffer (Beyotime, Shanghai, China) for 20 min, and then blocked with blocking buffer (Beyotime, Shanghai, China) for 30 min. After removal of the blocking solution, cells were incubated with primary antibodies at 4 °C overnight. After washing with PBS buffer 3 times, the cells were incubated with anti-rabbit Alexa Fluor 488-labeled secondary antibodies (Beyotime, Shanghai, China) and anti-mouse Alexa Fluor 555-labeled secondary antibodies (Beyotime, Shanghai, China) under protection from light at room temperature for 1 h. The cells were mounted in anti-fade mounting medium (Beyotime, Shanghai, China). Stained samples were inspected using confocal microscopy (Olympus, Tokyo, Japan).

### 4.10. RFP–GFP–LC3 Assay

HepG2 and L02 cells were seeded in confocal dishes at a density of 4 × 10^4^ cells/mL and incubated for 12 h. Cells were transfected with viral particles expressing RFP–GFP–LC3 (Invitrogen, Carlsbad, CA, USA), in accordance with the instructions of the manufacturer. After 24 h of transfection, cells were treated with TSN (10 µM), Baf (0.5 µM), or Rapa (1 µM) for 6 h. The colocalization of GFP and RFP puncta was imaged using confocal microscopy.

### 4.11. Transmission Electron Microscopy

HepG2 cells were treated with TSN (10 µM) over a certain time course (0, 6, 12 h) and then 2.5% glutaraldehyde in 0.1 M phosphate buffer (pH 7.4) was used for fixed cell sample. After 2 h, cells were dehydrated in a graded ethanol series and then embedded. Ultrathin sections of these cells were mounted on copper grids and then stained. Finally, transmission electron microscopy (Hitachi Ltd. HT7800/HT7700 Serial, Tokyo, Japan) was used for visualizing.

### 4.12. LTR Staining

HepG2 and L02 cells were treated with TSN (10 µM), Baf (0.5 µM), or Rapa (1 µM) for 6 h and then were incubated with 50 nM LTR (Invitrogen, Carlsbad, CA, USA) at 37 °C for 20 min. The cells were washed with PBS and then immediately observed under a confocal microscope. The LTR fluorescence intensity was quantified by ImageJ software.

### 4.13. DQ-BSA Assay

HepG2 and L02 cells were firstly incubated with 10 µg/mL DQ-BSA (Invitrogen, Carlsbad, CA, USA) in EBSS (Gibco, Grand Island, NY, USA) medium for 1.5 h and then treated with TSN (10 µM), Baf (0.5 µM), E64D (25 µM) plus pepstatin A (50 µM), EBSS for another 6 h. The cells were washed with PBS and then immediately observed under a confocal microscope. The DQ-BSA fluorescence intensity was quantified by ImageJ software.

### 4.14. CTSB Activity Assay

HepG2 cells were treated with TSN for 6 h at different concentrations (2.5, 5, 10, 20 µM). CTSB activity was assayed using a CTSB Activity Assay Kit (Fluorometric) (Abcam, #ab65300), in accordance with the instructions of the manufacturer. The mixtures of cell lysate, reaction buffer, and substrate were incubated for 2 h at 37 °C, and then fluorescent intensity (excitation 400 nm, emission 505 nm) was detected using a Multifunctional Microplate Reader (Biotek, Synergy H1, Winooski, VT, USA).

### 4.15. Animal Experiment

Thirty-six specific pathogen-free (SPF) male Balb/c mice (5–7 weeks) were obtained from the Laboratory Animal Center of Sun Yat-sen University in Guangzhou, China. The animal study was performed in accordance with the protocol approved by the Animal Ethical and Welfare Committee of Sun Yat-sen University (Approval No.: SYSU-IACUC-2021-000292). All mice were housed at 20–26 °C and 40–70% humidity, with a 12 h cycle of artificial lighting and free access to water and food. The mice were assigned randomly into four groups (*n* = 9 in each group). Mice in the vehicle group were treated with saline containing 10% propylene glycol, and the TSN groups were treated with different doses of TSN (5, 10, 20 mg/kg) dissolved in saline containing 10% propylene glycol through intraperitoneal injection. After treatment for 24 h, all mice were weighed and euthanized. Livers and blood were collected. Serum was isolated from blood for the determination of ALT, AST, and LDH levels. Livers were photographed, weighed, and then stored at −80 °C for subsequent analysis.

### 4.16. Serum Biochemical Assay

The serum levels of ALT, AST, and LDH were measured by an automatic analyzer (Hitachi Ltd. 3100 Serial, Tokyo, Japan) using standardized commercially available kits (Guangzhou Donglin Biotechnology Co., Guangzhou, China).

### 4.17. Histopathological Examinations (HE)

The livers were individually excised and immediately immersed in a formaldehyde solution for 24 h. The recipe for formaldehyde solution is 10% of 37–40% formaldehyde solution and 90% of 0.01 mol/L pH 7.4 PBS. After fixation and paraffin-embedding, 3-µm-thick sections were cut and stained with hematoxylin and eosin for overall morphological evaluation using an optical microscope (Nikon ECLIPSE E100, Nikon, Japan).

### 4.18. IHC

Livers were embedded in paraffin and sectioned into 3 µm thick slices. Immunohistochemistry was performed using antigen retrieval with high temperature treatment in Tris/EDTA buffer (pH 9.0). Anti-LC3B, anti-P62, and anti-CTSB antibodies were used as primary antibodies, and the secondary antibody was HRP-labeled. All specimens were examined by a microscope (Nikon ECLIPSE E100, Nikon, Japan).

### 4.19. Statistical Analysis

Data analysis was performed by Prism 7.0 (GraphPad Software, CA, USA). All data are expressed as the mean ± standard deviation (SD) of at least three independent experiments. Data analysis was determined using the Student’s two-tailed *t*-test or one-way ANOVA. *p* < 0.05 was considered statistically significant.

## 5. Conclusions

Our study demonstrated that TSN had significant hepatotoxicity both in vivo and in vitro. TSN suppressed hepatocyte autophagic flux by inhibiting TFEB expression and, thus, the lysosomal acidity, degradation function, and expression of hydrolase CTSB. Activation of autophagy could effectively alleviate the hepatotoxicity induced by TSN, indicating that autophagy has a protective effect against TSN-induced liver injury, which is expected to be further studied to develop an effective strategy for alleviating TSN-induced hepatotoxicity.

## Data Availability

Data is contained within the article or Supplementary Material.

## Supplementary Materials

The following supporting information can be downloaded at: https://www.mdpi.com/article/10.3390/ph15121509/s1, Figure S1: The mRNA levels of autophagy related genes.; Table S1: Real-time PCR primers sequences.

## Abbreviations

ALT	serum alanine aminotransferase
AST	aspartate aminotransferase
ATG	autophagy-related gene
Baf	bafilomycin A1
CLEAR	coordinated lysosomal enhancement and regulation
CQ	chloroquine
CTSB	cathepsin B
CTSD	cathepsin D
CYP	cytochromes
DILI	drug-induced liver injury
DQ-BSA	Dye Quenched-Bovine Serum Albumin
GFP	green fluorescent protein
GSH	glutathione
HDS	herbal medicine and dietary supplements
LAMP1	lysosomal membrane proteins 1
LAMP2	lysosomal membrane proteins 2
LC3	microtubule-associated proteins 1A/1B light chain 3
LDH	lactate dehydrogenase
LTR	LysoTracker Red
MTORC1	mechanistic target of rapamycin kinase complex 1
Rapa	rapamycin
RFP	red fluorescent protein
TFEB	transcription factor EB
TSN	toosendanin
