# Supplementary material for: Toosendanin Induces Hepatocyte Damage by Inhibiting Autophagic Flux via TFEB-Mediated Lysosomal Dysfunction
Source: Pharmaceuticals (Basel). 2022 Dec 3;15(12):1509. doi: 10.3390/ph15121509 (PMC9781622; doi:10.3390/ph15121509)
Supplement: Supplementary file 1 [file pharmaceuticals-15-01509-s001.zip › pharmaceuticals-1997369-supplementary.pdf]

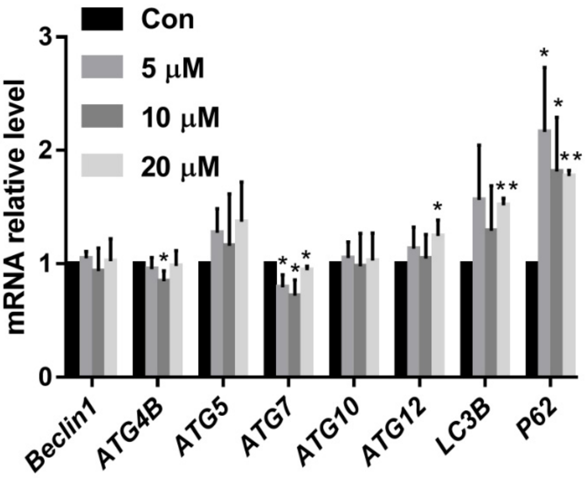

Supplemental Figure S1. The mRNA levels of autophagy related genes. HepG2 cells were treated with TSN for 6 h at different concentrations (5, 10, 20 μM). The mRNA levels of *Beclin1*, *ATG4B*, *ATG5*, *ATG7*, *ATG10*, *ATG12*, *LC3B* and *P62* was analyzed by quantitative real-time PCR. GAPDH was used as an internal control. Data are presented as the mean ± S.D. of three independent experiments. *p < 0.05, **p < 0.01 versus control.

Supplemental Table S1. Real-time PCR primers sequences

Gene	Forward sequence (5'-3')	Reverse sequence (5'-3')
Beclin1	CTGGACACTCAGCTCAACGTCA	CTCTAGTGCCAGCTCCTTTAGC
ATG4B	ATGGGAGTTGGCGAAGGCAAGT	AGCTCCACGTATCGAAGACAGC
ATG5	GCAGATGGACAGTTGCACACAC	GAGGTGTTTCCAACATTGGCTCA
ATG7	AGCTGAACGAGTATCGGCTG	GGTGGGAGCACTCATGTCAA
ATG10	TGGGAATGGAGACCATCAAAGG	GTAGCTCGAAAGCCTCCTCC
ATG12	GGGAAGGACTTACGGATGTCTC	AGGAGTGTCTCCCACAGCCTTT
LC3B	TTCGAGAGCAGCATCCAACC	GATTGGTGTGGAGACGCTGA
P62	TGTGTAGCGTCTGCGAGGGAAA	AGTGTCCGTGTTTCACCTTCCG
GAPDH	TGCACCACCAACTGCTTAGC	GGCATGGACTGTGGTCATGAG
